# Retraction: Specific blockade of Rictor-mTOR association inhibits mTORC2 activity and is cytotoxic in glioblastoma

**DOI:** 10.1371/journal.pone.0291490

**Published:** 2023-09-08

**Authors:** The PLOS ONE Editors

Following the publication of this article [1, 2], concerns were raised with results presented in Fig 1. Specifically:

- The S6K panel in Fig 1F of [1] appears similar to the Fig 2A U87_PTEN_ actin panel published in [3].
- The AKT panel in Fig 1F of [1] appears similar to the Fig 2A U87_PTEN_ p-AKT panel published in [3] when horizontally flipped.

The corresponding author commented that the above concerns were the result of errors made during figure preparation and stated that the underlying data for these panels are no longer available. In the absence of the original data underlying the published results these issues cannot be resolved. The corresponding author regrets any inconvenience to the scientific community that may have occurred, and requested the retraction of the article.

In light of the unresolved concerns with Fig 1 that question the reliability of these results, the *PLOS ONE* Editors retract this article.

MEJ and JG agree with retraction. ABS, JL, BH, KAL, TB, and AL either did not respond directly or could not be reached.
